# Aerobic Capacity Is Not Associated with Most Cognitive Domains in Patients with Multiple Sclerosis—A Cross-Sectional Investigation

**DOI:** 10.3390/jcm7090272

**Published:** 2018-09-11

**Authors:** Martin Langeskov-Christensen, Søren Eskildsen, Egon Stenager, Henrik Boye Jensen, Helle Hvilsted Nielsen, Thor Petersen, Lars Grøndahl Hvid, Päivi Hämäläinen, Lisbet Marstrand, Ulrik Dalgas

**Affiliations:** 1 Section for Sport Science, Department of Public Health, Aarhus University, 8000 Aarhus C, Denmark; soerenneskildsen@gmail.com (S.E.); lhvid@ph.au.dk (L.G.H.); dalgas@ph.au.dk (U.D.); 2 Department of Regional Health Research, University of Southern Denmark, Odense, Denmark/MS-Clinic of Southern Jutland (Sønderborg, Kolding, Esbjerg), Department of Neurology, Hospital of Southern Denmark, 6400 Sønderborg, Denmark; Egon.Stenager3@rsyd.dk; 3 Brain and Nerve Diseases, Department of Neurology, Hospital Lillebaelt, 6000 Kolding, Denmark; Henrik.Boye.Jensen@rsyd.dk; 4 The Multiple Sclerosis Clinic, Department of Neurology, Odense University Hospital, 5000 Odense C, Denmark; Helle.Hvilsted.Nielsen@rsyd.dk; 5 The Multiple Sclerosis Clinic, Department of Neurology, Aarhus University Hospital, 8000 Aarhus C, Denmark; thorpete@rm.dk; 6 Masku Neurological Rehabilitation Centre, 21250 Masku, Finland; paivi.hamalainen@neuroliitto.fi; 7 The Danish Multiple Sclerosis Centre, Department of Neurology, Rigshospitalet, 2100 Copenhagen, Denmark; lisbet.marstrand@regionh.dk

**Keywords:** aerobic training, exercise therapy, cognitive performance, cardiorespiratory fitness, VO_2_-max, maximal oxygen consumption

## Abstract

(1) Background: Cognitive impairment is highly prevalent in multiple sclerosis (MS). Staying physically fit may be associated with preservation of cognitive performance in persons with MS (pwMS); (2) Objective: To investigate the association between aerobic capacity and the cognitive domains of information processing, learning and memory, and verbal fluency as well as single and composite z-scores of the Brief Repeatable Battery of Neuropsychological tests (BRBNT) in pwMS; (3) Methods: All subjects first performed the BRBNT and then a maximal oxygen consumption (VO_2_-max) test on a bicycle ergometer as a measure of aerobic capacity. Simple and multiple (adjusting for age, sex, and education level) regression analyses were performed to evaluate the relationship between aerobic capacity and cognitive performance in different domains. Published international norms were used to compute z-scores for each individual and composite BRBNT score. Furthermore, cognitive impairment was defined as one or more z-scores ≤−1.5 standard deviation (SD) of healthy controls; (4) Results: Eighty-four subjects were included (44.9 ± 9 years, 16.3 ± 2 education years, Expanded Disability Status Scale (EDSS): 2.6 ± 1.4, MS-type (relapsing-remitting, primary progressive, or secondary progressive): 73/6/5, disease duration: 9.9 ± 7 years, VO_2_-max: 28.4 ± 7.0 mL O_2_/min/kg). No significant associations between aerobic capacity and cognitive performance in the individual BRBNT tests were found, except that a weak relationship was found between aerobic capacity and the composite processing speed z-score (R^2^ = 0.06, *p* = 0.02). The average global BRBNT z-score (−0.2 ± 0.66) was not associated with aerobic capacity. Comparison of the cognitively impaired group (34.5%) with the nonimpaired group (65.5%) showed lower aerobic capacity in the impaired group (25.9 ± 1 vs. 29.7 ± 1 mLO_2_/min/kg, *p* = 0.02); (5) Conclusions: Limited support was found for an association between performance in most cognitive domains and aerobic capacity in the present MS group with a third of patients showing signs of cognitive impairments.

## 1. Introduction

Multiple sclerosis (MS) is a chronic autoimmune and neurodegenerative disorder of the central nervous system. The progressively disabling nature of MS manifests as sensory, motor and cognitive impairments [1]. Cognitive impairment is reported in 35–60% of persons with MS (pwMS) and is often presented as slowed cognitive processing speed and impaired learning and memory [2]. Such impairments have been associated with unemployment, social isolation, reduced driving ability, and reduced quality of life in PwMS [2,3]. To counteract these consequences, several disease-modifying treatments and cognitive enhancing medications have been examined [4], but so far there are no approved pharmacological treatments for cognitive impairment in MS. Additionally, evidence regarding the efficacy of cognitive rehabilitation interventions for pwMS is limited [5]. Effective interventions preserving or improving cognitive performance are therefore warranted.

Aerobic exercise represents a promising approach towards possibly preserving and/or restoring cognitive performance in both pwMS and other populations. Systematic reviews and meta-analyses demonstrate that aerobic exercise improves overall cognitive performance in older adults and people with mild cognitive impairment [6,7,8,9]. To provide the best possible guidance for future randomised controlled trials investigating the effects of aerobic exercise on cognition in MS, cross-sectional investigations of associations between aerobic capacity and cognitive domains are important. However, the relationship between aerobic capacity and different cognitive performance domains remains inconclusive in pwMS, as studies have demonstrated both significant and nonsignificant correlations between aerobic capacity and processing speed [10,11,12,13,14], while few studies have investigated the association between aerobic capacity and executive functioning [15], memory and learning [10,12], and verbal fluency [12]. Of note, most existing studies are small sample size studies that have applied few single domain cognitive tests rather than a comprehensive validated battery of neuropsychological tests covering all major cognitive domains [16]. Furthermore, the existing studies also failed to compare cognitive performance of pwMS to norms of healthy controls and evaluated results by univariate correlations rather than by multiple regression analyses. By doing so significant confounders such as age, sex, and education level were not taken into account. This is problematic since an in-depth understanding of the association between aerobic capacity and cognitive performance may provide valuable insight into treatment targets, and subsequently, how to optimally design future studies on aerobic training and cognitive performance in pwMS.

Consequently, the aims of the present study were to investigate the association between aerobic capacity and the cognitive domains of information processing, learning and memory, and verbal fluency as well as single and composite z-scores of the Brief Repeatable Battery of Neuropsychological tests (BRBNT) in pwMS using both simple and multiple regression analyses.

We hypothesised that aerobic capacity would be positively associated with cognitive performance in the domain of information processing and a global composite z-score of the BRBNT.

## 2. Materials and Methods

The present cross-sectional study represents a secondary analysis of baseline data from an ongoing randomised, controlled trial investigating the effects of aerobic exercise on brain health and cognition in pwMS. This trial is registered at ClinicalTrials.gov (NCT02661555).

### 2.1. Subjects

Eighty-six subjects were recruited from Danish MS clinics at Aarhus University Hospital, Viborg Regional Hospital and the MS Clinic of Southern Jutland. They were invited (but not selected), at consultations, to participate in the primary study comprising a long-term exercise intervention. Inclusion criteria were 18–65 years, a definite MS diagnosis according to the McDonald criteria [17], Expanded Disability Status Scale (EDSS) score ≤6.0, and ability to transport oneself back and forth from the test and training facility. Exclusion criteria were dementia (ICD10 diagnosis or dementia symptoms in hospital journals), comorbidities preventing participation (e.g., cardiovascular disease or metabolic diseases), pregnancy, alcohol abuse, pacemaker, relapse within 8 weeks before inclusion, or participation in systematic aerobic training (i.e., moderate to high intensity training more than twice per week) prior to inclusion. All subjects gave their written informed consent for inclusion before they participated in the study. The study was conducted in accordance with the Declaration of Helsinki, and the protocol was approved by the Ethics Committee of Region Midtjylland (1-10-72-291-15).

**Figure 1:**
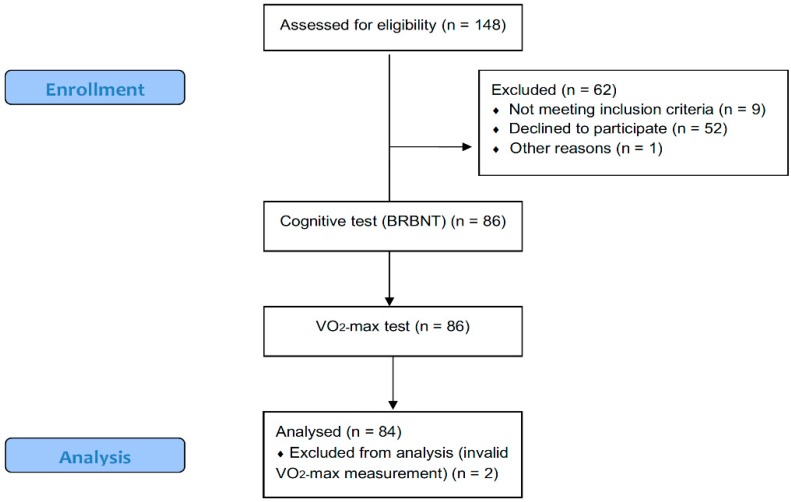
Subject enrolment and analysis.

After inclusion, subjects were scheduled to individually perform the BRBNT followed by a maximal oxygen consumption (VO_2_-max) test. Both tests were performed in the morning and on the same day and were supervised by one of three investigators who were all trained to perform both the BRBNT and the VO_2_-max test. A study overview is presented in Figure 1.

### 2.2. Cognitive Testing

The BRBNT [16], which was specifically developed to examine cognitive performance in pwMS, was applied, in a Danish version, to test cognitive performance in specific cognitive domains. The BRBNT contains seven different tests which were conducted in the following order. (1) The Selective Reminding Test (SRT), a measure of verbal learning consisting of both the Long-term Retrieval Test (SRT-L) and the Consistent Long-term Retrieval Test (SRT-C); (2) the 10/36 Spatial Recall Test (SPART), a measure of visuospatial learning; (3) the Symbol Digit Modalities Test (SDMT), a measure of sustained attention and information processing speed; (4) the Paced Auditory Serial Additional Test (PASAT; 3-second version), a measure of sustained attention and information processing speed; (5) the Word List Generation Test (WLG), a verbal fluency test; (6) the Delayed Recall of the SRT (SRT-D); and (7) the Delayed Recall of the SPART (SPART-D). For all tests, the higher the score, the better the cognitive performance. Further details on how to perform these tests have been described elsewhere [18]. The cognitive tests were performed in a quiet room without any disturbances. The cognitive test battery was manually performed and assessed on paper, and the results of each test were electronically entered, stored, and compared by two researchers to avoid typing errors. The objective of each test in the BRBNT is listed in Table 1.

In order to compare the cognitive performance of pwMS to norms of healthy controls, z-scores for each individual cognitive test were computed based on published norms from a sample of 140 healthy Dutch subjects [18]. This comparison was conducted to evaluate whether cognitive impairment had a significant impact on the association between aerobic capacity and cognitive performance. Consistent with other neuropsychological studies [19,20], subjects were classified as cognitively impaired if one or more of the individual BRBNT z-scores were ≥−1.5 standard deviation (SD) unit below the mean of published norms [18]. Furthermore, to investigate whether composite cognitive performance scores would correlate with aerobic capacity, composite z-scores were calculated for each cognitive domain: z-verbal memory (Z_v_), z-visual memory (Z_vi_), z-processing speed (Z_ps_), z-fluency (Z_f_), and for the entire BRBNT (z-global (Z_global_). These scores were calculated as [21],

Z_v_ = (z SRT-L + z SRT-C + z SRT-D)/3 (1)

Z_vi_ = (z SPART + z SPART-D)/2 (2)

Z_ps_ = (z SDMT + z PASAT)/2 (3)

Z_f_ = z WLG (4)

Z_global_ = (Z_v_ + Z_vi_ + Z_ps_ + Z_f_)/4 (5)

### 2.3. Aerobic Capacity

Subjects performed an incremental exercise test until exhaustion on a bicycle ergometer (SRM, Jülich, Germany). The test was conducted at a self-chosen cadence between 55 and 95 revolutions per minute with an initial 5-minute warm up at 40 W followed by increments of 10 W/min (women) or 15 W/min (men) until voluntary exhaustion. Based on the expected maximal power output determined based on age, gender, disability, and body size, individual power output adjustments were made immediately after the 5-minute warm up in order to exhaust the subjects within 8 to 12 min after warm up [22]. Expired gas was collected in a mixing bag. The rate of oxygen uptake (VO_2_), carbon dioxide release (VCO_2_), and respiratory exchange ratio (RER) was determined continuously by an online respiratory gas exchange analyser (Oxigraf O2CPX, Oxigraf Inc., Sunnyvale, CA, USA) and expressed as 10-second averages using Indoor 8.00 software (Innovision ApS, Glamsbjerg, Denmark). Heart rate (HR) was monitored with a Polar watch (Polar A300, Oulu, Finland) and work rate (W) was monitored continuously using SRM software (SRM, Jülich, Germany). Prior to each test, the flow and gas analysers were calibrated using a 3 L syringe (Hans Rudolph, series 5530, Shawnee, KS, USA) and certified reference gasses (4.00% CO_2_ and 16.5% O_2_). Subjects were told to refrain from eating 2 h before testing but were allowed to drink water ad libitum before and after the test. Saddle and handlebars were adjusted to match each subject’s anthropometrics, and subjects were verbally encouraged to continue the test as long as possible. The subjects were asked to rate their perceived exhaustion (RPE) after voluntary exhaustion using the 6–20 Borg scale [23]. Body mass and fat percentage were collected prior to each VO_2_-max test, using a calibrated personal scale (Tanita SC-330, Tokyo, Japan). The highest recorded 30-second VO_2_ average that was attained during the test was considered the VO_2_-max. It has previously been shown that an incremental VO_2_-max test is a reliable and valid method for testing VO_2_-max in mildly to moderately impaired pwMS [24]. Aerobic capacity was expressed as mL O_2_/kg bodyweight/min based on the VO_2_-max test.

### 2.4. Statistical Analyses

All data was checked for normality by visual inspection of histograms and QQ-plots. Then, a univariate regression analysis was performed to evaluate the crude associations between aerobic capacity and the different cognitive performance tests (SRT-L, SRT-C, SRT-D, SPART, SPART-D, SDMT, PASAT, WLG, and all z-scores). Next, a multiple regression analysis was applied, adjusting for known potential confounding factors (age, sex, and education years). The selected predictor variables were selected a priori, were based on clinical relevance and identified in cooperation with a neuropsychologist. The level of education was expressed as the total number of years of formal schooling, as reported by the subjects. In all multiple regression analyses cognitive performance was considered the dependent variable. The regression coefficients were interpreted to evaluate clinical relevance. Moreover, differences in all parameters between the cognitive performance subgroups were examined using independent t-tests as well as ANCOVA for aerobic capacity (adjusting for age, sex, and education years). All statistical analyses were performed using Stata version 15 (StataCorp LP, College Station, Texas, TX, USA). Significance level was set at *p* ≤ 0.05.

## 3. Results

### 3.1. Subject Characteristics

Characteristics and descriptive data on the outcome measures are presented in Table 2 and Table 3. Wide ranges of age, disability (EDSS), disease duration (defined as years since diagnosis), and aerobic capacity (body-weight-normalised VO_2_-max) were observed. Two subjects were excluded; one due to invalid VO_2_-max measurements and one due to an incomplete VO_2_-max test. Hence, baseline results from 84 subjects remained for the analyses. 

### 3.2. Simple Linear Regression Analyses

The simple linear regression analyses revealed that aerobic capacity was not associated with any of the BRBNT tests of cognitive performance (Table 4). A trend was seen towards an association between aerobic capacity and SDMT (*p* = 0.06) and PASAT (*p* = 0.07).

### 3.3. Multiple Linear Regression Analyses

After adjusting for age, sex and education years, the association between aerobic capacity and cognitive performance remained nonsignificant for all BRBNT tests (Table 5). Age was a significant covariate in all cognitive performance scores except the PASAT and the WLG. Sex was a significant covariate in SRT-L, SRT-C, SRT-D, and WLG. Education years was a significant covariate only in the PASAT. The multivariate regression analyses provided significant models for predicting scores of all cognitive test scores, except the PASAT. 

### 3.4. Z-Scores of the BRBNT 

**Figure 2:**
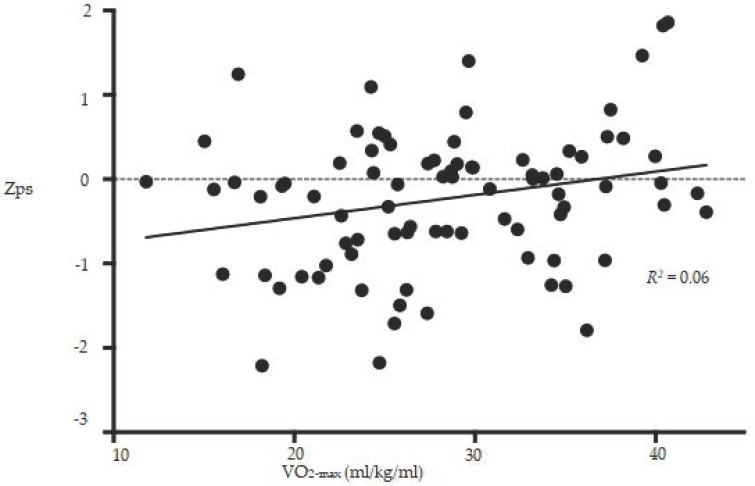
Association between Z_ps_ and aerobic capacity. The association was not significant when adjusted for age, sex, and education years. Z_ps_: Composite z-score for the Paced Auditory Serial Additional Test (PASAT) and the Symbol Digit Modalities Test (SDMT), VO_2_-max: Maximal oxygen consumption.

The mean Z_global_ was −0.2 ± 0.66 SD. Simple and adjusted regression analyses showed no significant associations between aerobic capacity and Z_global_, Z_verbal_, Z_visual_, and Z_fluency_. A significant, but weak, association was found between aerobic capacity and Z_ps_ in the simple, but not in the multiple, regression analysis (regression coefficient 0.03 ± 0.01, *p* = 0.02, Figure 2).

Twenty-nine subjects (34.5%) were classified as cognitively impaired, and when compared with the 55 pwMS in the nonimpaired group (65.5%), the cognitively impaired group had lower aerobic capacity (25.9 ± 1 vs. 29.7 ± 1 mLO_2_/min/kg, *p* = 0.02) (Table 2) as well as lower scores in all tests of the BRBNT (Table 3). When adjusted for age, sex, and education years this difference remained (*p* = 0.04). 

## 4. Discussion

The main finding of the present study was that aerobic capacity does not (SRT, SRT-L, SRT-C, SPART, SDMT, PASAT, WLG, SRT-D, SPART-D, Z_global_, Z_verbal_, Z_visual_, Z_fluency_) or only to a very limited extent (Z_ps_) explain variance in cognitive performance tests in pwMS. Importantly, the study applied a validated battery of neuropsychological tests and multiple regression analyses accounting for age, sex and education level in an MS sample covering a broad range of disabilities (EDSS 0–6), ages (23–64 years), and cognition levels (nonimpaired to impaired).

### 4.1. Effects on Information Processing

Results are somewhat promising regarding the association between aerobic capacity and the cognitive domain of information processing speed when summarising the existing MS literature (please see Table A1 for a full overview of previous studies). Two studies by Prakash et al. found significant associations between aerobic capacity and information processing speed assessed by the PASAT (*r* = 0.42) [12] and a composite processing speed score (SDMT, PASAT, and WLG; *r* = 0.45) [14]. In accordance, three studies by Sandroff et al. reported comparable significant associations between aerobic capacity and the SDMT (*r* = 0.41) [10], the Modified Flanker task (*r* = −0.62) [13] and a composite processing speed score (PASAT and SDMT; *r* = 0.44) [11]. Though only weak to moderate, the associations reported by Prakash et al. and Sandroff et al. differs from the present study in that they did not find significant associations between aerobic capacity and the PASAT or SDMT. However, the association between aerobic capacity and the composite Z_ps_ was significant, albeit weak, in the present study. The discrepancy to previous studies may relate to the study designs, as all previous studies included cognition as the primary outcome (but not based on a power analysis) as opposed to the present study. Nevertheless, the present study enrolled more participants than the previous studies, making this difference between studies less likely to explain the different findings. Like the present study, none of the previous studies involved a priori recruitment of cognitively impaired pwMS or recruited subjects based on being low-fit. Furthermore, aerobic capacity was measured using the gold standard testing (i.e., a respiratory gas exchange analyser and an incremental exercise test until exhaustion) in all studies, and education level was similar in the studies reporting number of education years [12,13,14]. These similarities support direct comparison between studies. Conversely, the studies by Prakash et al. only included females and two out of the three studies by Sandroff et al. predominantly included females (87–93.8% vs. 58% in the present study) complicating a direct comparison.

### 4.2. Effects on Memory and Learning

The present study confirms previous studies in MS [10,12] by not finding any associations between aerobic capacity and the cognitive domain of memory and learning. In a small study comprising 24 females, Prakash et al. found a partial correlation, adjusted for age, education, and disease duration, of −0.22 between aerobic capacity and SRT-L [12]. However, this finding may be biased by the adjustment for several covariates in a small sample size and should therefore be interpreted cautiously [25]. Sandroff et al. reported a somewhat similar correlation of 0.19 in the domain of memory and learning, but this study applied a different validated test (California Verbal Learning Test (CVLT-2)) [10] than the one used in the present study, not allowing a direct comparison. Prakash et al. and Sandroff et al. both reported nonsignificant correlation coefficients of 0.12 (assessed by the SPART) [12] and 0.18 (assessed by the Brief Visuospatial Memory Test-Revised (BVMT-R)) when focusing on visuospatial function [10]. These findings are in line with those of the present study, suggesting no association between aerobic capacity and cognitive performance in the domain of memory and learning in pwMS.

### 4.3. Effects on Verbal Fluency

The cognitive domain of verbal fluency has previously been investigated in two studies by Prakash et al. [12,14], of which only one reported the outcome. The WLG, a measure of semantic retrieval, was not associated with aerobic capacity in that study [12]. Similarly, the present study found no association between these outcomes, suggesting that aerobic capacity does not influence verbal fluency in pwMS.

### 4.4. Cognitively Impaired vs. Nonimpaired

The present sample of pwMS attained a global z-score of −0.2 ± 0.66 SD lower than that of healthy Dutch controls, indicating an overall cognitively well-functioning sample. This could have led to potential ceiling effects in the BRBNT. Additionally, the low z-score underlines that for the present study the pwMS were not included based on the presence of cognitive impairment. Moreover, the fact that the participants signed up for a long-term exercise intervention may have caused a selection bias by making most people with severe cognitive impairments unwilling to join the study. This might have caused the low presence of cognitive impairment in this sample (34.5%) when compared to previous studies (up to 60%) [2]. However, the rate of cognitively nonimpaired/impaired also relies heavily on the applied definition of cognitive impairment. The present study applied one definition for cognitive impairment (i.e., having one or more of the individual BRBNT z-scores below −1.5 SD of healthy controls) that was consistent with previous studies on the topic [20]. The applied definition is obviously debatable, but it offers a more conservative discrimination than most other definitions that can be found. Finally, potential effects of disease modifying therapies must also be considered when interpreting the relatively low rate of cognitive impairment in the present sample. In the present study 82% of the pwMS used disease modifying therapies which is comparable to previous related studies (79%) [10].

Aerobic capacity in the cognitively impaired and cognitively nonimpaired subgroups was compared in subgroup analyses. The cognitively impaired subjects had a significantly lower aerobic capacity (25.9 ± 6.7 vs. 29.7 ± 7.3 mLO_2_/min/kg), also after adjusting for age, sex, and education level, indicating a better aerobic capacity in cognitively nonimpaired pwMS. Although this diverges from the findings of the regression analyses, the difference in aerobic capacity may be attributed to the applied categorization of cognitive impairment which may have caused a polarised representation of cognitively impaired pwMS (i.e., by having one or more of the individual BRBNT z-scores below −1.5 SD of healthy controls).

### 4.5. Direction of Causality

The direction of causality in the observed associations cannot be elucidated based on the present cross-sectional study. It remains unclear whether aerobic capacity affects processing speed or vice versa, or whether a bidirectional association exists. In order to unravel this, longitudinal exercise intervention studies were located. To date, ten randomised controlled trials have investigated the effects of different exercise modalities on cognitive performance in the domain of information processing in pwMS [26,27,28,29,30,31,32,33,34,35]. However, the majority of these have several methodological concerns including reliance upon unsupervised training interventions, low-intensity exercise prescription (with no subsequent physical improvement following the intervention), and low-quality methods for measuring physical function. Only the study by Kucuk et al. found a positive effect, after 8 weeks of pilates exercise, on information processing speed (PASAT; 3-second version) compared with a control condition [29]. However, the control group intervention was not described in detail [29], making it challenging to interpret the findings. In an exploratory study Kierkegaard et al. found positive effects on the SDMT after 12 weeks of high-intensity resistance training [35] but these results are not comparable since distinctive physiological effects are expected following resistance training vs. endurance exercise [36]. Moreover, learning effect significantly contributes to continuous improvement in the SDMT [37]. The studies by Briken et al. [26] and Zimmer et al. [34] managed to provide and measure significant improvements in aerobic capacity after aerobic exercise interventions but no concurrent effects could be detected in the SDMT. However, significantly improved verbal learning and memory were demonstrated in both studies. These findings contrast the results of the present study where aerobic capacity and memory and learning were not associated. This comparison is, however, greatly influenced by the differences in study populations with subjects in the present study being substantially less physically and cognitively disabled.

In summary, the longitudinal studies regarding the impact of exercise on cognitive performance provide some insight into the direction of causality (i.e., exercise may impact cognition) but results are still contradictory. The interpretation of previous studies is furthermore blurred by the use of diverse methodologies comprising different exercise modalities (intensity, type, and duration), varying cognitive assessments (single tests vs. validated batteries) and dissimilar groups.

### 4.6. Potential Underlying Mechanisms 

Previous studies have shown positive effects of aerobic exercise on cognitive performance in healthy [38], elderly [9,39], and MS populations [26,29,33,34]. As a consequence, several underlying mechanisms have been proposed in pwMS. One such mechanism relates to exercise induced changes in insulin-like growth factor (IGF-1) and brain-derived neurotrophic factor (BDNF). BDNF likely plays a role in neuronal survival and activity-dependent plasticity and studies indicate that exercise could promote this factor in pwMS [40,41], whereas animal studies have shown that IGF-1 appears to act as a neuroprotective agent through increased uptake of circulating IGF-1 by the brain [42]. Exercise has also been shown to upregulate hippocampal BDNF which may play a role in lessening the decline in cognitive performance associated with MS [42]. However, whether these alterations could alter the course of MS, or in this case the development in cognitive performance in pwMS, remains hypothetical.

Another potential underlying mechanism involves an (aerobic) exercise-mediated increase in cerebral blood flow (CBF). It has been demonstrated that aerobic capacity is associated with improved levels of CBF dynamics in healthy persons [43]. Furthermore, a recent review by El-Sayes et al. states that exercise-induced increases in CBF is a mediator of neural connectivity by providing neurons with necessary nutrients such as oxygen and glucose [44]. Increased CBF during acute exercise furthermore allows IGF-1 and vascular endothelial growth factor (VEGF) produced in other tissues to cross the blood–brain barrier which, when combined (also with BDNF produced in the brain [45]), over time may promote structural and functional brain changes leading to improved cognitive performance [44,46]. However, this model of neuroplasticity induced by repeated bouts of aerobic exercise remains to be proven in MS.

Finally, whole brain atrophy, which occurs at a faster rate in pwMS compared to healthy controls (0.5–1% vs. 0.1–0.3% yearly), has been proposed as a potential mechanism linking exercise to cognitive performance [47]. Longitudinal magnetic resonance imaging (MRI) studies have shown strong associations between changes in cognitive performance and increases in brain atrophy [48]. Also, brain atrophy progression early in the disease is able to predict cognitive impairment 5 years later [48]. However, more specific MRI findings on T2-lesion load, decreased deep and cortex grey matter volumes, and impairments of white matter integrity have also been associated with a reduction in cognitive performance; particularly information processing speed [49,50,51]. Interestingly, structural MRI in a study by Colcombe et al. revealed that physical fitness is related to preservation of brain volume in older adults [52], and that 6 months of aerobic exercise produces significant increases in brain volume compared to a stretching and toning control group [53]. However, this is opposed in a similar study by Jonasson et al. showing neutral findings in cortical thickness (yet with marked improvements in aerobic capacity) [39]. In pwMS, Prakash et al. found that aerobic capacity was associated with regional grey matter volumes [14], suggesting a protective effect on brain volume with increasing levels of aerobic capacity. Furthermore, Kjølhede et al. recently demonstrated that 24 weeks of progressive resistance training tended to reduce whole brain atrophy in pwMS when compared to a habitual lifestyle in the control group [54]. Accordingly, there is preliminary evidence for a prophylactic influence of exercise on the structural decline of brain tissue in pwMS which in turn may affect cognitive performance. However, much more knowledge on this topic is needed before sound conclusions can be made. 

### 4.7. Limitations

Several limitations must be kept in mind when interpreting the present study. First, given the nature of the cross-sectional design, no conclusions can be made on the direction of causality. Second, cognitive performance was a secondary outcome in a large randomised, controlled trial. Thus, the study was potentially underpowered, which could have resulted in type II errors. However, the present study enrolled more participants than previous studies on this topic. Third, the prospect of participating in a long-term randomised, controlled trial may have discouraged pwMS with more severe cognitive impairment from participating, and encouraged pwMS being more positive towards exercise, which may affect the generalisability of the results (i.e., only one third of the sample were cognitively impaired). Furthermore, lack of subjects with severe cognitive impairment may have weakened the associations to aerobic capacity. Fourth, z-scores were computed based on internationally published norms from The Netherlands [18]. It is recommended to compare cognitive performance with national norm data to minimize language, education, or cultural differences, but no Danish norm data exist on the BRBNT. Adding some justification to the applied procedure, Sepulcre et al. compared BRBNT norm values from Spain, The Netherlands, and Italy and found similar results, indicating little influence of language [21]. Fifth, the studied subjects represent a subset of pwMS with mild to moderate disabilities (EDSS from 0.0 to 6.0). Therefore, the associations found in the present study may not apply to more severely impaired pwMS or to pwMS with progressive types of the disease (this study only included 11 subjects with progressive MS). 

## 5. Conclusions

The present study provides limited support for an association between performance in most cognitive domains and aerobic capacity in pwMS with relatively preserved cognitive capacity. Further studies including well-defined cognitively impaired pwMS and a validated battery of neuropsychological tests are needed to enlighten the direct effects of exercise on cognitive performance in pwMS.

## Figures and Tables

**Table 1:** Objective and primary cognitive domain of each neuropsychological test in the Brief Repeatable Battery of Neuropsychological tests (BRBNT).

Cognitive Test	Measures of:	Cognitive Domain
SRT (including SRT-L, SRT-C & SRT-D)	Verbal learning and memory	Memory and learning
SPART (including SPART-D)	Visuospatial learning and delayed recall	Memory and learning
SDMT	Sustained attention and information processing speed	Information processing
PASAT	Sustained attention and information processing speed	Information processing
WLG	Semantic retrieval	Verbal fluency

SRT-L: Selective Reminding Test-Long-term Storage, SRT-C: Selective Reminding Test-Consistent Long-term Retrieval, SRT-D: Selective Reminding Test-Delayed recall, SPART: 10/36 Spatial Recall Test, SPART-D: 10/36 Spatial Recall Test-Delayed Recall, SDMT: Symbol Digital Modalities Test, PASAT: Paced Auditory Serial Addition Test, WLG: Word List Generation.

**Table 2:** Subject characteristics.

	Total Sample	Range	CI Group	CN Group
Sex (men/women)	35/49		15/14	20/35
Age (years)	44.9 ± 9.4	(23–64)	48.8 ± 8.1	42.9 ± 9.4 *
Height (cm)	173.9 ± 10.0	(154–198)	174.3 ± 10.9	173.7 ± 9.6
Weight (kg)	76.0 ± 16.8	(46–153)	74.3 ± 16.2	77.0 ± 17.2
Fat percent (%)	28.4 ± 9.1	(10–47)	25.7 ± 8.4	29.8 ± 9.3 *
BMI (kg/m^2^)	25.0 ± 4.2	(17–39)	24.2 ± 3.8	25.4 ± 4.3
VO_2_-max (L/min)	2.13 ± 0.6	(0.9–3.6)	1.9 ± 0.7	2.2 ± 0.5 *
Aerobic Capacity (ml O_2_/min/kg)	28.4 ± 7.3	(11–42)	25.9 ± 6.7	29.7 ± 7.3 *
RER (VCO_2_/VO_2_)	1.21 ± 0.08	(1.01–1.39)	1.20 ± 0.09	1.21 ± 0.08
HR_max_ (beats/min)	168 ± 15.8	(124–200)	162.7 ± 15.7	171.4 ± 15.1 *
RPE	17.2 ± 1.4	(13–20)	17.0 ± 1.5	17.4 ± 1.3
Education (years)	16.5 ± 2.8	(10–25)	15.1 ± 2.8	17.2 ± 2.5 *
DMT use (yes/no)	69/15		21/8	48/7
EDSS	2.67 ± 1.44	(0–6)	3.2 ± 1.4	2.4 ± 1.4 *
MS type (RR, PP, SP)	73/6/5		24/3/2	49/3/3
Disease duration (years)	9.9 ± 7.1	(0–38)	11.2 ± 8.2	9.3 ± 6.4

Values are means ± SD, (range) or number of subjects. CI: Cognitively impaired, CN Cognitively nonimpaired, VO_2_-max: Peak oxygen consumption, RER: Respiratory Exchange Ratio, HR_max_: Maximal Heart Rate, RPE: Rating of Perceived Exhaustion, DMT: Disease Modifying Treatment EDSS: Expanded Disability Status Scale, RR: Relapsing-Remitting, PP: Primary progressive, SP: Secondary Progressive. * CN significantly different from the CI group.

**Table 3:** Results of the BRBNT cognitive test battery.

Test (Score Range)	Total Sample	Range	CI Group	CN Group
SRT-L (0–72)	47.2 ± 15.0	(0–69)	36.9 ± 17.7	52.7 ± 9.8 *
SRT-C (0–72)	37.2 ± 16.8	(0–69)	25.6 ± 15.9	43.3 ± 13.9 *
SRT-D (0–12)	8.9 ± 2.6	(3–12)	7.0 ± 2.6	10.0 ± 2.0 *
SPART (0–30)	22.1 ± 5.3	(11–30)	18.6 ± 5.2	24.0 ± 4.3 *
SPART-D (0–10)	7.6 ± 2.2	(3–10)	6.1 ± 2.1	8.4 ± 1.8 *
SDMT (0–110)	55.7 ± 14.9	(33–110)	47.1 ± 9.4	60.2 ± 15.4 *
PASAT (0–60)	43.0 ± 13.0	(0–60)	34.2 ± 15.2	47.7 ± 8.7 *
WLG (0–∞)	27.5 ± 7.6	(13–51)	22.4 ± 7.0	30.3 ± 6.5 *

Values are means ± SD and (range). CI: Cognitively impaired, CN: Cognitively nonimpaired, SRT-L: Selective Reminding Test-Long-term storage, SRT-C: Selective Reminding Test-Consistent long-term retrieval, SRT-D: Selective Reminding Test-Delayed recall, SPART: 10/36 Spatial Recall Test, SPART-D: 10/36 Spatial Recall Test-Delayed recall, SDMT: Symbol Digital Modalities Test, PASAT: Paced Auditory Serial Addition Test, WLG: Word List Generation. * CN significantly different from the CI group.

**Table 4:** Simple linear regression coefficients between aerobic capacity and cognitive performance.

Test	Coefficient (SEM)	*p*-Value	*R*^2^
SRT-L	0.17 (0.22)	0.44	0.01
SRT-C	0.28 (0.25)	0.26	0.02
SRT-D	0.03 (0.04)	0.47	0.01
SPART	0.05 (0.08)	0.49	0.01
SPART-D	0.00 (0.03)	0.95	0.00
SDMT	0.42 (0.22)	0.06 ^#^	0.04
PASAT	0.35 (0.19)	0.07 ^#^	0.04
WLG	0.13 (0.11)	0.24	0.01

^#^: Borderline significant association. SEM: Standard Error of the Mean. SRT-L: Selective Reminding Test-Long-term storage, SRT-C: Selective Reminding Test-Consistent long-term retrieval, SRT-D: Selective Reminding Test-Delayed recall, SPART: 10/36 Spatial Recall Test, SPART-D: 10/36 Spatial Recall Test-Delayed recall, SDMT: Symbol Digital Modalities Test, PASAT: Paced Auditory Serial Addition Test, WLG: Word List Generation.

**Table 5:** Multiple linear regression coefficients between aerobic capacity and cognitive performance, adjusted for sex, age, and education years.

Test	Variable	Coefficient (SEM)	*p*-Value	*R*^2^
SRT-L	Aerobic capacity	0.14 (0.21)	0.52	0.27 ** (*p* ≤ 0.001)
	Age	−0.56 (0.16)	0.001 *	
	Sex	7.08 (3.10)	0.03 *	
	Education years	1.03 (0.54)	0.06	
SRT-C	Aerobic capacity	0.24 (0.23)	0.24	0.33 ** (*p* ≤ 0.001)
	Age	−0.66 (0.17)	<0.001 *	
	Sex	8.60 (3.34)	0.01 *	
	Education years	1.32 (0.58)	0.03 *	
SRT-D	Aerobic capacity	0.02 (0.04)	0.52	0.31 ** (*p* ≤ 0.001)
	Age	−0.10 (0.27)	<0.001 *	
	Sex	1.40 (0.53)	0.01 *	
	Education years	0.17 (0.09)	0.06	
SPART	Aerobic capacity	0.23 (0.08)	0.77	0.19 ** (*p* = 0.001)
	Age	−0.16 (0.06)	0.001 *	
	Sex	1.25 (1.16)	0.28	
	Education years	0.43 (0.20)	0.03 *	
SPART-D	Aerobic capacity	−0.01 (0.03)	0.70	0.16 ** (*p* = 0.02)
	Age	−0.06 (0.02)	0.01 *	
	Sex	−0.37 (0.48)	0.44	
	Education years	0.17 (0.08)	0.04 *	
SDMT	Aerobic capacity	0.33 (0.22)	0.13	0.24 ** (*p* ≤ 0.001)
	Age	−0.65 (0.16)	<0.001 *	
	Sex	3.54 (3.16)	0.27	
	Education years	0.17 (0.55)	0.75	
PASAT	Aerobic capacity	0.32 (0.20)	0.12	0.10 (*p* = 0.09)
	Age	−0.01 (0.15)	0.92	
	Sex	0.63 (2.98)	0.83	
	Education years	1.11 (0.52)	0.03 *	
WLG	Aerobic capacity	0.19 (0.11)	0.10	0.21 ** (*p* = 0.001)
	Age	−0.15 (0.08)	0.08	
	Sex	5.46 (1.64)	0.001 *	
	Education years	0.27 (0.28)	0.34	

*: Statistically significant covariate. **: Statistically significant model. SEM: Standard Error of the Mean. SRT-L: Selective Reminding Test-Long-term storage, SRT-C: Selective Reminding Test-Consistent long-term retrieval, SRT-D: Selective Reminding Test-Delayed recall, SPART: 10/36 Spatial Recall Test, SPART-D: 10/36 Spatial Recall Test-Delayed recall, SDMT: Symbol Digital Modalities Test, PASAT: Paced Auditory Serial Addition Test, WLG: Word List Generation.

## Appendix A

**Table A1:** Overview of studies presenting cross-sectional correlations between aerobic capacity and cognitive performance.

Study/Domain	Memory and Learning	Information Processing	Executive Function	Verbal Fluency
Prakash (2007) n = 24	SRT: *r* = −0.22 SPART: *r* = 0.12	SDMT: *r* = 0.17, PASAT: *r* = 0.42 *	-	WLG: *r* = 0.30
Prakash (2010) n = 21	Not shown	Composite Z_ps_ score (PASAT, SDMT, WLG): *r* = 0.46 *	-	Not shown
Sandroff (2012) n = 31	-	Composite Z_ps_ score: PASAT, SDMT: *r* = 0.44 *	-	-
Sandroff (2015) n = 28	-	-	Modified flanker task: Congruent and Incongruent Reaction time: *r* = −0.54 * *r* = −0.48 *	-
Sandroff (2015) n = 62	CVLT-2: *r* = 0.19 BVMT-R: *r* = 0.18	SDMT *r* = 0.41 *	-	-
Sandroff (2017) n = 64	-	SDMT: Not shown Modified flanker task: *r* = −0.28 *	-	-

*: Significant correlation (*p* < 0.05). -: Not measured. Not shown: assessed but not shown.
