# Correction: Langeskov-Christensen, M., *et al*. Aerobic Capacity Is Not Associated with Most Cognitive Domains in Patients with Multiple Sclerosis—A Cross-Sectional Investigation. *Journal of Clinical Medicine* 2018, *7*, 272

**DOI:** 10.3390/jcm8050574

**Published:** 2019-04-26

**Authors:** Martin Langeskov-Christensen, Søren Eskildsen, Egon Stenager, Henrik Boye Jensen, Helle Hvilsted Nielsen, Thor Petersen, Lars Grøndahl Hvid, Päivi Hämäläinen, Lisbet Marstrand, Ulrik Dalgas

**Affiliations:** 1 Section for Sport Science, Department of Public Health, Aarhus University, 8000 Aarhus C, Denmark; soerenneskildsen@gmail.com (S.E.); lhvid@ph.au.dk (L.G.H.); dalgas@ph.au.dk (U.D.); 2 Department of Regional Health Research, University of Southern Denmark, Odense, Denmark/MS-Clinic of Southern Jutland (Sønderborg, Kolding, Esbjerg), Department of Neurology, Hospital of Southern Denmark, 6400 Sønderborg, Denmark; Egon.Stenager3@rsyd.dk (E.S.); Henrik.Boye.Jensen@rsyd.dk (H.B.J.); 3 Brain and Nerve Diseases, Department of Neurology, Hospital Lillebaelt, 6000 Kolding, Denmark; 4 The Multiple Sclerosis Clinic, Department of Neurology, Odense University Hospital, 5000 Odense C, Denmark; Helle.Hvilsted.Nielsen@rsyd.dk; 5 The Multiple Sclerosis Clinic, Department of Neurology, Aarhus University Hospital, 8000 Aarhus C, Denmark; thorpete@rm.dk; 6 Masku Neurological Rehabilitation Centre, 21250 Masku, Finland; paivi.hamalainen@neuroliitto.fi; 7 The Danish Multiple Sclerosis Centre, Department of Neurology, Rigshospitalet, 2100 Copenhagen, Denmark; lisbet.marstrand@regionh.dk

The authors wish to make the following corrections to this paper [1].

The authors have identified a statistical error regarding the number of cognitively impaired patients since incorrect standard deviations of the healthy reference population were unfortunately used when calculating the z-scores of the individual cognitive tests; the correct number of patients categorized as cognitively impaired therefore has to be adjusted from 29 (34.5%) to 37 (44.0%). Due to this error, all affected statistical analyses were performed again. As a consequence of this change, the cognitively impaired group (CI group) no longer had a lower aerobic capacity when compared to the cognitively nonimpaired group (CN group). All other results were unchanged. Hence, this finding is now in line with the overall findings of the regression analyses. Moreover, it has no impact on the conclusions of our paper.

Accordingly,

**Table 2:** Subject characteristics.

	Total Sample	Range	CI Group	CN Group
Sex (men/women)	35/49		15/14	20/35
Age (years)	44.9 ± 9.4	(23–64)	48.8 ± 8.1	42.9 ± 9.4 *
Height (cm)	173.9 ± 10.0	(154–198)	174.3 ± 10.9	173.7 ± 9.6
Weight (kg)	76.0 ± 16.8	(46–153)	74.3 ± 16.2	77.0 ± 17.2
Fat percent (%)	28.4 ± 9.1	(10–47)	25.7 ± 8.4	29.8 ± 9.3 *
BMI (kg/m^2^)	25.0 ± 4.2	(17–39)	24.2 ± 3.8	25.4 ± 4.3
VO_2_-max (L/min)	2.13 ± 0.6	(0.9–3.6)	1.9 ± 0.7	2.2 ± 0.5 *
Aerobic Capacity (ml O_2_/min/kg)	28.4 ± 7.3	(11–42)	25.9 ± 6.7	29.7 ± 7.3 *
RER (VCO_2_/VO_2_)	1.21 ± 0.08	(1.01–1.39)	1.20 ± 0.09	1.21 ± 0.08
HR_max_ (beats/min)	168 ± 15.8	(124–200)	162.7 ± 15.7	171.4 ± 15.1 *
RPE	17.2 ± 1.4	(13–20)	17.0 ± 1.5	17.4 ± 1.3
Education (years)	16.5 ± 2.8	(10–25)	15.1 ± 2.8	17.2 ± 2.5 *
DMT use (yes/no)	69/15		21/8	48/7
EDSS	2.67 ± 1.44	(0–6)	3.2 ± 1.4	2.4 ± 1.4 *
MS type (RR, PP, SP)	73/6/5		24/3/2	49/3/3
Disease duration (years)	9.9 ± 7.1	(0–38)	11.2 ± 8.2	9.3 ± 6.4

Values are means ± SD, (range) or number of subjects. CI: Cognitively impaired, CN Cognitively nonimpaired, VO_2_-max: Peak oxygen consumption, RER: Respiratory Exchange Ratio, HR_max_: Maximal Heart Rate, RPE: Rating of Perceived Exhaustion, DMT: Disease Modifying Treatment EDSS: Expanded Disability Status Scale, RR: Relapsing-Remitting, PP: Primary progressive, SP: Secondary Progressive. * CN significantly different from the CI group.

should be replaced with

**Table 2:** Subject characteristics.

	Total Sample	Range	CI Group	CN Group
Sex (men/women)	35/49		19/18	16/31
Age (years)	44.9 ± 9.4	(23–64)	47.5 ± 8.8	42.9 ± 9.4 *
Height (cm)	173.9 ± 10.0	(154–198)	174.6 ± 10.9	173.4 ± 9.3
Weight (kg)	76.0 ± 16.8	(46–153)	74.4 ± 14.9	77.3 ± 18.2
Fat percent (%)	28.4 ± 9.1	(10–47)	26.0 ± 8.9	30.3 ± 9.0 *
BMI (kg/m^2^)	25.0 ± 4.2	(17–39)	24.3 ± 3.7	25.6 ± 4.5
VO_2_-max (L/min)	2.13 ± 0.6	(0.9–3.6)	2.0 ± 0.7	2.2 ± 0.6
Aerobic Capacity (ml O_2_/min/kg)	28.4 ± 7.3	(11–42)	26.9 ± 6.7	29.5 ± 7.6
RER (VCO_2_/VO_2_)	1.21 ± 0.08	(1.01–1.39)	1.21 ± 0.08	1.21 ± 0.08
HR_max_ (beats/min)	168 ± 15.8	(124–200)	164.6 ± 14.2	171.4 ± 16.5 *
RPE	17.2 ± 1.4	(13–20)	17.1 ± 1.4	17.4 ± 1.4
Education (years)	16.5 ± 2.8	(10–25)	15.2 ± 2.6	17.5 ± 2.5 *
DMT use (yes/no)	69/15		28/9	41/6
EDSS	2.67 ± 1.44	(0–6)	3.1 ± 1.4	2.4 ± 1.4 *
MS type (RR, PP, SP)	73/6/5		31/3/3	42/3/2
Disease duration (years)	9.9 ± 7.1	(0–38)	10.6 ± 8.5	9.4 ± 5.8

Values are means ± SD, (range) or number of subjects. CI: Cognitively impaired, CN Cognitively nonimpaired, VO_2_-max: Peak oxygen consumption, RER: Respiratory Exchange Ratio, HR_max_: Maximal Heart Rate, RPE: Rating of Perceived Exhaustion, DMT: Disease Modifying Treatment EDSS: Expanded Disability Status Scale, RR: Relapsing-Remitting, PP: Primary progressive, SP: Secondary Progressive. * CN significantly different from the CI group.

The authors apologize for any inconvenience caused to the readers by these changes.
